# Effects of Reduced pH on *Macoma balthica* Larvae from a System with Naturally Fluctuating pH-Dynamics

**DOI:** 10.1371/journal.pone.0068198

**Published:** 2013-06-26

**Authors:** Anna Jansson, Joanna Norkko, Alf Norkko

**Affiliations:** 1 Environmental and Marine Biology, Åbo Akademi University, Åbo, Finland; 2 Tvärminne Zoological Station, University of Helsinki, Hanko, Finland; 3 Marine Research Centre, Finnish Environment Institute, Helsinki, Finland; National Institute of Water & Atmospheric Research, New Zealand

## Abstract

Ocean acidification is causing severe changes in the inorganic carbon balance of the oceans. The pH conditions predicted for the future oceans are, however, already regularly occurring in the Baltic Sea, and the system might thus work as an analogue for future ocean acidification scenarios. The characteristics of the Baltic Sea with low buffering capacity and large natural pH fluctuations, in combination with multiple other stressors, suggest that OA effects may be severe, but remain largely unexplored. A calcifying species potentially affected by low pH conditions is the bivalve *Macoma balthica* (L.). We investigated larval survival and development of *M. balthica* by exposing the larvae to a range of pH levels: 7.2, 7.4, 7.7 and 8.1 during 20 days in order to learn what the effects of reduced pH are on the larval biology and thus also potentially for the population dynamics of this key species. We found that even a slight pH decrease causes significant negative changes during the larval phase, both by slowing growth and by decreasing survival. The growth was slower in all reduced pH treatments compared to the control treatment. The size of 250 µm that is considered indicative to imminent settling in our system was reached by 22% of the larvae grown in control conditions after 20 days, whereas in all reduced pH treatments the size of 250 µm was reached by only 7–14%. The strong impact of ocean acidification on larvae is alarming as slowly growing individuals are exposed to higher predation risk in response to the longer time they are required to spend in the plankton, further decreasing the ecological competence of the species.

## Introduction

Anthropogenic emissions of carbon dioxide (CO_2_) are causing severe changes in the global inorganic carbon balance of the oceans [1]. Approximately one third of the atmospheric CO_2_ is absorbed in the oceans due to the pCO2 difference in ocean surface and atmosphere. This removal of CO_2_ from the atmosphere will help moderate future climate change [2], however, the reaction of excess CO_2_ in the seawater has severe effects on marine ecosystems [3], [4]. A direct consequence of CO_2_-induced ocean acidification (OA) is the reduction in carbonate ion concentration (CO_3_^2−^). This makes calcification, the building of calcium carbonate structures, one of the biological processes most sensitive to ocean acidification. A decrease in the calcification rate in response to reduced pH level and CaCO_3_ saturation state has already been found in several marine species [3], [5], [6]. Bivalve early life-stages are generally considered susceptible to acidification [7], [8] with impacts such as delayed and/or abnormal development [9]–[11], slower growth [11]–[14], reduced calcification [13] and higher mortality [10], [12], [14]. Responses to ocean acidification are, however, species-specific; differences in tolerance have been observed even between closely related taxa [7], [8]. Predicting the impact of OA on the early life stages is thus essential for estimating the true consequences of future and ongoing ocean acidification [15]. A reduced performance of the early-life stages is alarming as larvae are crucially important for sustaining viable populations, and a failure in their recruitment might ultimately lead to negative effects on the population.

An example of a species potentially affected by low pH conditions is the bivalve *Macoma balthica* (L.). In the Baltic Sea, *M. balthica* is regarded as a key species. It is abundant throughout the Baltic Sea, often dominating biomass in soft sediments from organic mud to sandy bottoms at a variety of depths, from the very shallow down to 190 m [16]–[20]. It is an important prey organism, and has a central role in sediment reworking and bioturbation, contributing to the overall health and functioning of the benthic ecosystem [21]. In the species-poor northern Baltic Sea, no other species in the current community can fully replace its important functions [22]. In these areas, *M. balthica* lives in salinity as low as 5, which is close to the species salinity tolerance limit. Living in a stressful environment such as in an oligohaline environment or at the edge of the distribution range causes energetic constraints that can be seen as slow growth rates, thin and weak shells and small size like in the case of many Baltic Sea bivalves [23]–[25]. During the pelagic larval phase, abundances of up to 12000 larvae/m^3^ are measured in the Baltic Sea [26].

The benthic communities of the Baltic Sea are living in an environment characterized by substantial riverine input and steep geographical and seasonal gradients in e.g. temperature, salinity, alkalinity and biological productivity. The ecosystem is exposed to a variety of stressors of anthropogenic origin, such as eutrophication, oxygen deficiency and pollution [27]. Large areas of the sea floor are permanently oxygen depleted, both in the deep areas of the open sea and in coastal areas [28]. The species living in these conditions are adapted to strong variation in e.g. salinity and temperature, but the exposure to multiple stressors might increase the sensitivity and susceptibility of benthic fauna towards additional disturbances such as OA. Ocean acidification is predicted to be severe in the Baltic Sea due to the naturally low buffering capacity of the system caused by low alkalinity [29], and a drop of up to 0.45 pH units has been estimated for the surface waters during this century (Omstedt et al. unpublished) [30]. Reliable and accurate predictions of the future pH levels are, however, generally hard to make for coastal areas with highly variable pH, and especially so for systems with low buffering capacity such as the Baltic Sea [30].

Natural pH variability in the Baltic Sea is already large, and the highest seasonal pH differences are found in the northern areas of the Baltic Sea, such as the Gulf of Finland and the Bothnian Sea [31]. Strong seasonal pH fluctuations occur mainly due to changes in primary production [31]–[33] or associated with seasonal hypoxia [34]. During peaks of high photosynthetic activity during phytoplankton blooms, the CO_2_ concentration in the water column exhibits substantial diurnal variation resulting in high pH values of up to 8.6 during daytime and low pH values such as 7.5 during respiration at night. Also vertical variation in pH is large with a steep decrease with depth; values as low as 7.2 occur regularly near the seafloor (1 m above the seabed, at a depth of 66 m at the station LL5 Finnish Environment Institute, unpublished data). OA is likely to increase pH fluctuations, making the occasionally experienced extreme pH levels even more pronounced and common, further adding to the stress the organisms have to cope with [31], [34], [35]. The effects of OA on benthic communities have, however, not been assessed before in the northern Baltic Sea [30].

The characteristics of the Baltic Sea, with low buffering capacity and large natural variations in pH, in combination with multiple other stressors, suggest that OA effects may be severe, but remain largely unexplored. The prediction is that early life-history stages in particular may be very sensitive to OA. To investigate the response of the key-species *M. balthica* to ocean acidification, we conducted an experiment to investigate larval survival and development when exposed to different pH levels. Our aim was to learn what the effects of reduced pH are on the larval biology and thus also potentially for the population dynamics of *M. balthica*.

## Materials and Methods

### Collecting of larvae

*M. balthica* spawning starts after water temperature has reached approximately 7°C in spring and continues until a temperature of about 14°C is reached [36]. The growth rates in the area are not well recorded, but the time spent in the plankton is generally longer than in fully marine systems (2–5 weeks [37]), and it is estimated that the duration of the planktonic phase could be >6 weeks long. The planktonic life stage ends when the individual has reached a sufficient developmental stage (including increased mobility of the foot) and size (shell length) to metamorphose and settle to the seafloor [37]. A majority of the very newly settled bivalves encountered in our system have a size of 250–300 µm [38]–[40]. Peak settling in the northern parts of the Baltic Sea typically occurs in July [26], [38]. During the planktonic phase, *M. balthica* larvae experience large pH fluctuations including very low pH levels. *M. balthica* has shells composed of aragonite calcite [41].

The sensitivity of *M. balthica* larvae to OA was tested in a laboratory experiment by exposing the larvae to a range of pH levels at Tvärminne Zoological Station in the southwestern Finnish archipelago in May 2011. Pelagic bivalve larvae were collected from a long-term monitoring site within a nature reserve owned by the University of Helsinki, with permit for research granted by Tvärminne Zoological Station (University of Helsinki). By regular samplings throughout the spring we found that the larvae started to appear in the water column ca. 10 days before the experiment start. The sampling of larvae was conducted using a 100-µm plankton net. The samples were taken from 15 m depth to the surface, and the total depth of the site was approximately 35 m. The larvae were concentrated by gently washing them through a series of nets in order to remove smaller as well as larger particles (detritus, zooplankton and phytoplankton).

### Experimental set-up

Water for the experiment came from an adjacent bay (10 m depth), and was filtered through a series of filters (100, 50, 5 µm). The experiment was conducted in a continuous flow-through system and was run for 20 days. In accordance with the large natural variability in the Baltic Sea pH, a wide range of pH levels was tested. In addition to the control value of an average 8.1, three reduced pH levels were chosen for the experimental manipulation: 7.7, 7.4 and 7.2. Mixing of the inflowing seawater to each desired pH level was carried out in header tanks (100 l), one for each treatment. Controlled CO_2_-addition to the header tank was acquired with a computerized control system (AquaMedic pH-computer, AB AquaMedic GmbH) attached to a CO_2_-gas bottle that released a suitable amount of pure gaseous CO_2_ into the tank in order to reach and maintain the desired pH-level. At the start of the experiment, pH was gradually decreased to each target level during 1-2 days. Water was continuously flowing (flow rate 45 ml/min) from each header tank to 5 replicate experimental beakers (3 l Erlenmeyer flasks) containing the larvae (4.2 larvae/ml) with the initial size of 151±10 µm. The larvae were fed daily to satiation with a commercial algae mix (Shellfish diet, Reed Mariculture US). With continuous flow of water, all treatment beakers remained saturated with oxygen.

### Sampling

The appropriate parameters for determining the components of the carbonate system were measured regularly throughout the experiment. Total alkalinity (TA) and dissolved inorganic carbon (DIC) samples were taken from each beaker and tank three times during the experiment: in the beginning, on day 13, and at the end of the experiment on day 21. TA was determined from filtered samples with potentiometric titration using an automated titrator at 25°C (VINDTA-program, Metrohm 716 DMS Titrino). Dissolved inorganic carbon (DIC) was analysed immediately after sampling using a carbon analyser [42]. Salinity was measured with a conductivity meter (VWR EC 300) on the above mentioned sampling points. pH was measured daily directly in the experimental beakers on a NBS-scale with PH1000 h pH-meter (VWR) with a precision of 0.001 units and double-checked with a Jenway 3510 pH-meter with a precision of 0.01 units. Other parameters of the carbonate system were calculated from TA and DIC using CO2SYS [43] with dissociation constants according to Roy et al. [44]. The number of larvae was quantified every two days. A 10 ml subsample was taken from each flask, live and dead individuals were counted under a dissecting microscope, and the sample conserved for later analysis (4% buffered formaldehyde). Larvae were photographed and growth was studied by measuring shell length under a microscope with an ocular micrometer (Leica Microsystems). Larval sizes at the end of the experiment were measured from 105 individuals from each treatment. These individuals were collected after removing the 10 ml subsample.

### Statistical analysis

To study the relationship between larval survival, pH treatment and time (day of the experiment), we constructed a generalized linear mixed model (GLMM) with a Poisson error distribution where time and pH treatment explained the abundance of larvae. We also included the two-way interaction between time and pH treatment. The model included replicate identity as a random effect to control for repeated observations of replicates, and was fitted using Laplace approximation. The model, however, suffered from a slight overdispersion. As no serious outliers, no zero inflation and no spatial correlation were detected, the model was accepted for use. Statistics for the overall interaction term were obtained by comparing a model without the interaction term and a model including the interaction term. This statistical analysis was performed in the software R (version 2.15.1; R Core Team 2012).

The size of the larvae in the different pH-treatments was compared using a chi-square test. For this analysis, sizes at the end of the experiment were divided into 6 size classes. The analysis was conducted using SPSS version 21. Differences were considered significant at p<0.05 for all tests. Data are presented as means ±SD and modes.

## Results

### Experimental conditions

**Figure 1:**
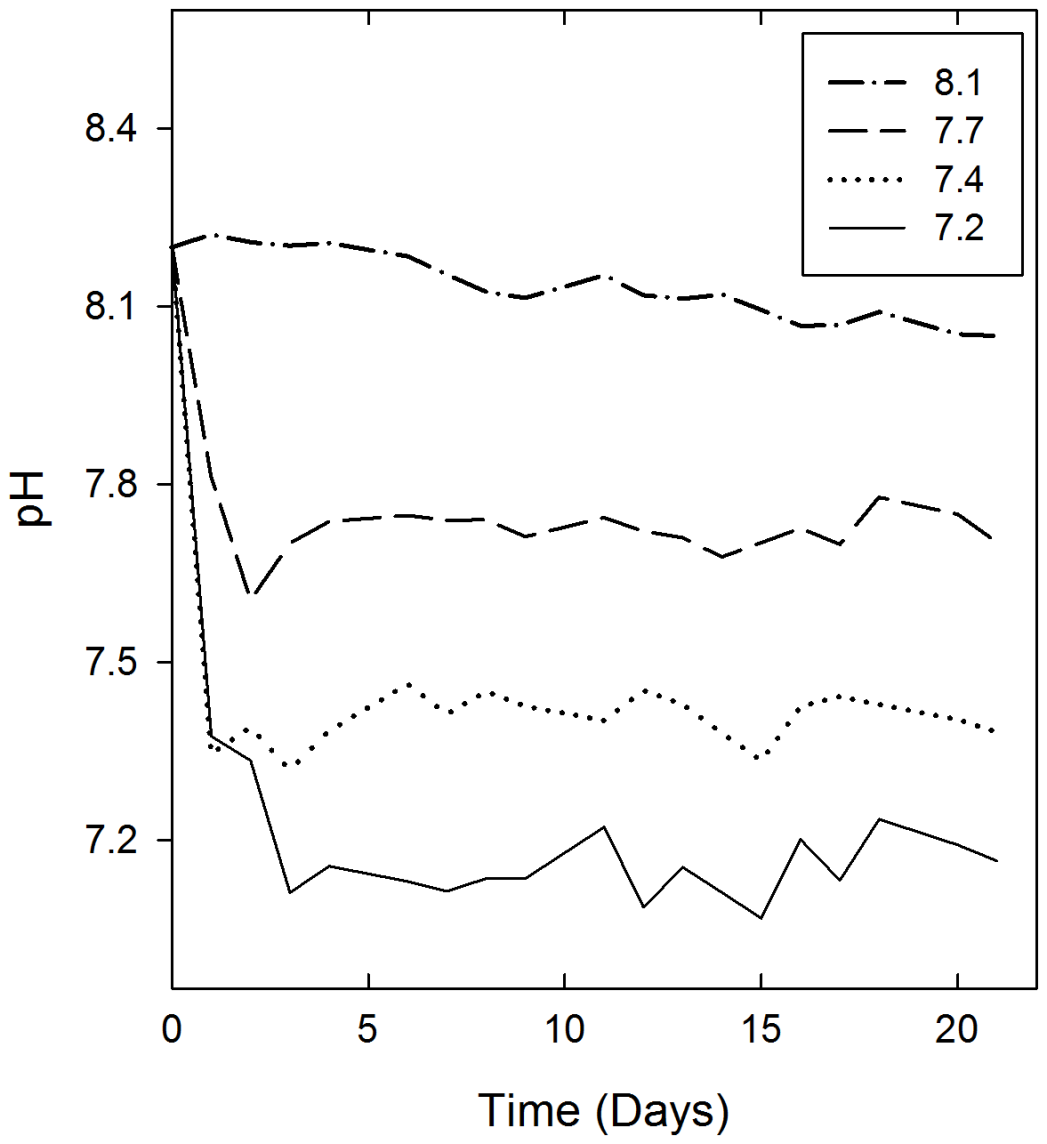
Mean pH levels during the experiment. Measured from all replicate bottles (n = 5) in each treatment. For clarity SD are not shown.

Salinity and total alkalinity fluctuated very little during the experiment (salinity 6.3±0.1, TA 1551±21 µmol/kg). Water temperature changed with natural conditions from 6°C at the start of the experiment to 12°C at the end. Dissolved oxygen level was over 10 mg/l in all treatments throughout the experiment. Treatment of seawater with CO_2_-addition resulted in DIC concentrations of an average 1498 (±26) µmol/kg for control pH_8.1_, 1556 (±21) µmol/kg for pH_7.7_, 1627 (±22) µmol/kg for pH_7.4_ and 1668 (±29) µmol/kg for pH_7.2_ (Table 1). Daily average pH values (±SD) for different treatments were 7.17 (±0.08), 7.40 (±0.04), 7.72 (±0.04) and 8.13 (±0.06) (Fig. 1). Although not specifically monitored, no indications of differences in day- and night time pH were observed.

**Table 1:** Carbonate system speciation for each pH treatment calculated from total alkalinity (TA) and dissolved inorganic carbon (DIC), mean ±SD.

	pH_7.2_	pH_7.4_	pH_7.7_	pH_8.1_
pH_NBS_	7.17±0.08	7.40±0.04	7.72±0.04	8.13±0.06
Salinity	6.3±0.1	6.3±0.1	6.3±0.1	6.3±0.1
TA (µmol/kg)	1548±22	1550±19	1554±17	1549±28
DIC (µmol/kg)	1688±29	1627±22	1556±21	1498±26
*p*CO_2_ (µatm)	4400±300	2800±280	1700±110	540±130
Ω_calc_	0.24±0.04	0.37±0.07	0.65±0.09	1.79±0.14
Ω_arag_	0.13±0.03	0.26±0.05	0.38±0.06	1.02±0.12

### Effects of reduced pH on larval survival and growth

**Figure 2:**
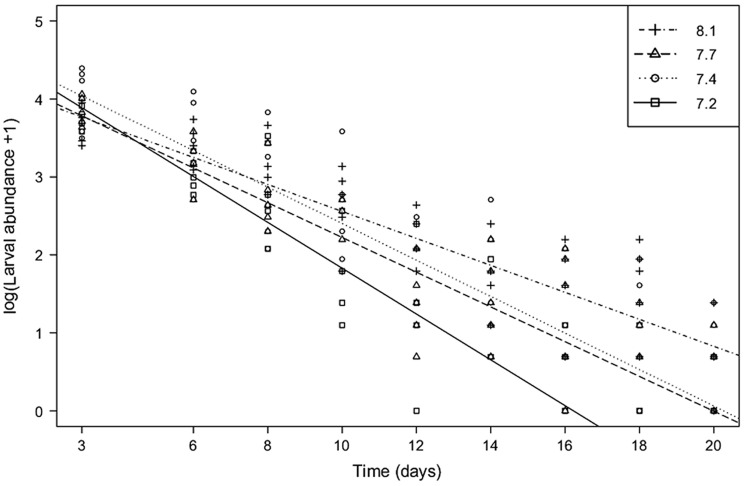
Larval abundance in different pH treatments over time. The relationship between logarithmic larval abundance and time for four different pH treatments. Lines were plotted using coefficients from the GLMM. The first two sampling points were on days 3 and 6, after which the larval sampling was conducted every other day. Each replicate (n = 20) for each sampling point is shown.

The larval abundance decreased in the control treatment with 95.2% to 0.2 larvae/ml during the 20 day-experiment, and the abundance of the larvae was even lower in low pH treatments: ca. 98% in pH_7.7_ and pH_7.4_ (to 0.12 and 0.14/ml) and 99.6% in pH_7.2_ (to 0.02/ml). While we found a general strong negative association between time and larval abundance, this association was modulated by pH treatment (chi square χ2 = 27.3, p<0.001, Fig. 2). The association between larval abundance and time was weakest in the control pH_8.1_ followed by stronger associations in pH_7.4_ and pH_7.7_. The strongest association between larval abundance and time was found in the treatment pH_7.2_ (Fig. 2, Table 2).

**Table 2:** Results of the GLMM (Poisson error distribution, log link function and replicate identity as a random factor).

	Estimate	Std. error	z-value	p-value
Intercept	4.769	0.207	23.039	**<0.001**
pH 7.4	−0.027	0.284	−0.095	0.924
pH 7.7	−0.317	0.285	−1.112	0.266
pH 8.1	−0.480	0.280	−1.718	0.086
Time	−0.294	0.019	−15.158	**<0.001**
pH 7.4 * time	0.060	0.025	2.438	**<0.05**
pH 7.7 * time	0.071	0.025	2.859	**<0.01**
pH 8.1 * time	0.121	0.024	5.136	**<0.001**

Effects of pH treatment and time on larval abundance.

All two-way interactions between time and pH treatment were significant and are denoted in bold.

**Figure 3:**
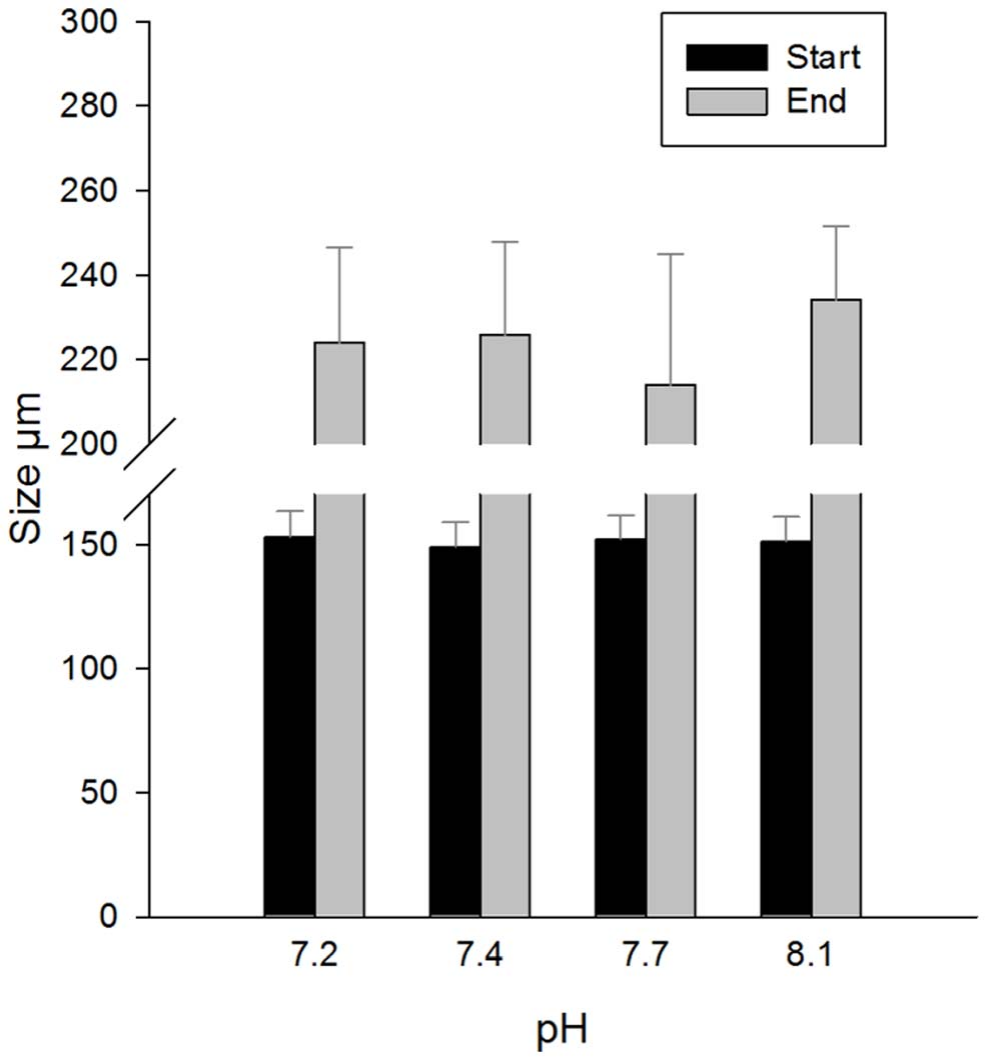
Larval sizes at the start and end of the experiment in different pH treatments. Mean ±SD, n = 105.

**Figure 4:**
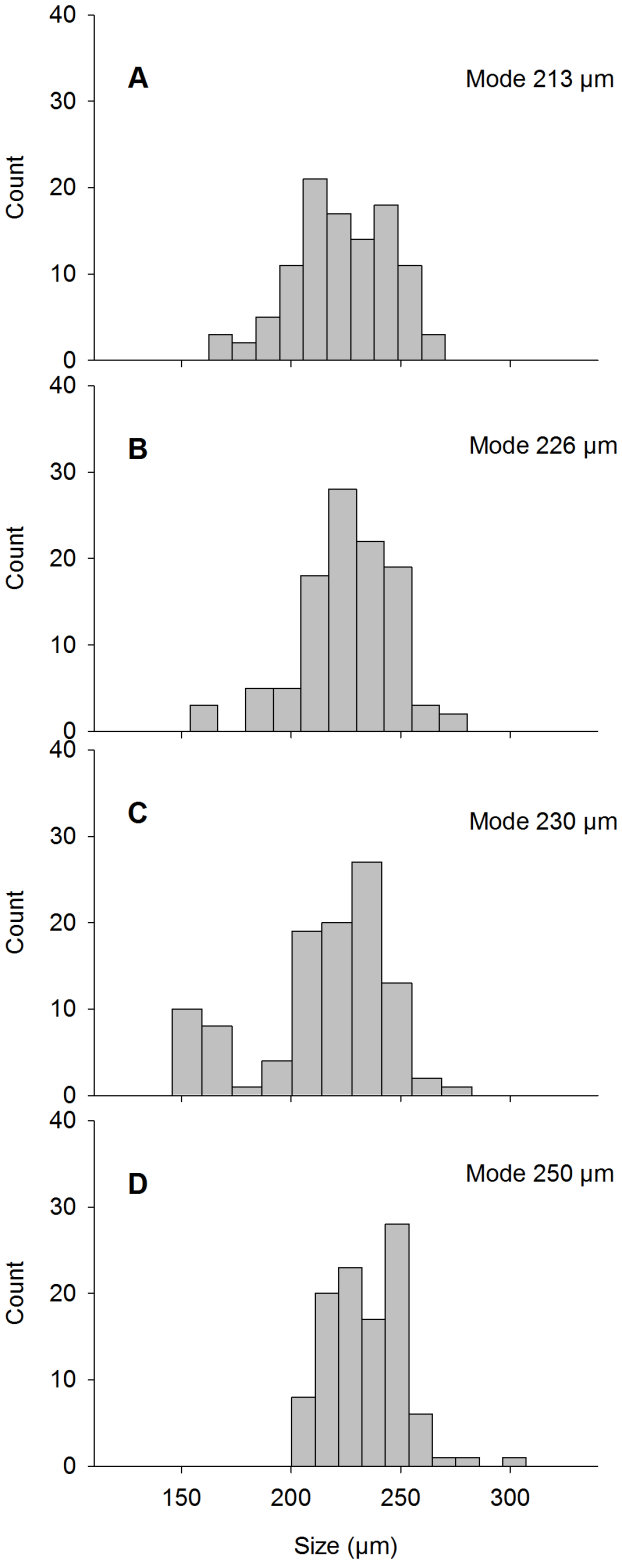
Larval sizes at the end of the 20-day experiment in different pH treatments. Larval sizes are illustrated as size frequency distributions in pH treatments (A) 7.2, (B) 7.4, (C) 7.7 and (D) 8.1, and presented with mode values illustrating the increase of large size fractions in the control treatment.

Larval size at the end of the experiment differed between pH treatments (chi-square χ^2^ = 48.6, p<0.001). Larvae in the control treatment reached an average size of 234±18 µm, whereas in pH treatments 7.2, 7.4 and 7.7, the average sizes were 224±23, 225±22 and 214±31 µm, respectively (Fig. 3). A large number of the larvae in the reduced pH-treatments showed a very limited growth, with minimum sizes of 125–130 µm in the beginning of the experiment compared to minimum sizes of 146–162 µm at the end of the experiment. Mode sizes decreased successively with pH, with lowest values in pH_7.2_ (213 µm) and highest in pH_8.1_ (250 µm; Fig. 4). Difference in average growth compared to the control treatment was: 14% less growth in the pH_7.2_, 10% less growth in the pH_7.4_, and 25% less growth in the pH_7.7_. The size indicative of imminent settling, 250 µm, was reached by 22% of the larvae grown in control conditions, and in the reduced pH treatments by only 7–14%.

## Discussion

pH of the global oceans is decreasing and a decrease of up to 0.5 pH units is estimated to occur by the year 2100 [3], [45]. In the Baltic Sea, seawater pH is naturally highly variable due to highly seasonal primary production and low alkalinity, and consequently, future oceanic pH levels are already at times measured in this area and a strong further decrease is estimated to occur due to the ongoing ocean acidification. This emphasizes the urgent need for information considering the effects of reduced pH on the communities in this area. We investigated larval survival and development of a bivalve, *M. balthica*, under low pH conditions in order to learn what the effects of reduced pH are on the larval biology and thus also potentially for the population dynamics of this key species. Our results show that the larvae are adversely affected by ocean acidification. Survival decreased with reduced pH over time and we also observed significant reductions in larval growth in low pH treatments, which affects subsequent ecological competence, i.e. any reduction in growth will weaken the potential for survival in the important post-larval stage [26].

Bivalve larvae that are exposed to low pH levels have previously been shown to be more sensitive to future acidification than many other organism groups [4], [46]. These responses are, however, species-specific, ranging from negative, to non-significant, to positive [7], [8]. Miller *et al*. [13] measured calcification in oyster larvae, and found a significant reduction in calcification rate and growth of larvae of *Crassostrea virginica* in high pCO_2_ regimes, but found no significant effects in another oyster species *Crassostrea ariakensis*. Crim *et al*. [10] discovered a 40–100% reduction in development in abalone larvae that were reared at pH_8.0_ and pH_7.7_ compared to the larvae reared in control conditions. Similar results were found by Kurihara *et al*. [11] who studied the effects of reduced pH on larval mussels, *Mytilus galloprovincialis*. *M. galloprovencialis* larvae that were exposed to pH_7.4_ during one week developed abnormalities and also grew significantly slower. Reduced growth of larvae of three bivalve species was also found by Talmage & Gobler [12] when clam, scallop and oyster larvae were grown in pH_7.85_ and pH_7.5_ for 18 days. The response of the larvae in our experiment is comparable to the response of *M. balthica* larvae in a system with higher alkalinity and hence buffering capacity towards pH fluctuations, the North Sea, where van Colen *et al*. [14] also detected high mortality, slow growth and delayed metamorphosis during the pelagic larval stage of *M. balthica* when exposed to pH_7.8_ and pH_7.5_.

We found significant pH-induced effects on the abundance of *M. balthica* larvae over time. High mortality was measured in all treatments, which is natural for early life-stages of invertebrates [26], [47], [48]. Importantly, even a slight change in their survivorship may have long-lasting consequences. During the planktonic phase, *M. balthica* larvae may encounter large pH fluctuations including very low pH values. Strong natural short-term pH fluctuations that occur in the Baltic Sea on a daily basis expose the bivalves to low pH levels already during early-life stages. We detected a 70–85% drop in larval abundance after 8–10 days exposure to pH_7.2_, but a similar sharp decrease in abundance in treatments 7.4 and 7.7 occurred later, after 10–12 days of exposure. A steady decrease of ca. 6% d^−1^ was observed in the control treatment until day 14. This indicates that strong pH fluctuations might cause an immediate effect, i.e. sudden drops may be detrimental. Future ocean acidification (in combination with continued eutrophication) will potentially exacerbate this by making the amplitude and frequency of the fluctuations even higher [31], [34]. High food level has been shown to counteract the effects of low pH on calcifying organisms [49], thus the on-going eutrophication in the Baltic Sea might initially balance the process. The interactive effects of multiple stressors such as eutrophication and OA are, however, complex and are currently not known, but they are likely to severely reduce the persistence of *M. balthica* populations.

Clear impacts of reduced pH levels on larval growth were observed. Size frequency distribution of the larvae was significantly skewed after the 20-day exposure to low pH levels. Moreover, larvae that were grown in low pH treatments showed large variation in sizes, with the largest variation in pH_7.7_. Small size fractions of larvae survived in the 7.7 treatment, with 21% of the measured larvae being smaller than 200 µm (Fig. 4). In comparison, in pH_7.2_ and pH_7.4_ the majority of the smallest size fractions were absent. The size of 250 µm was reached by 22% of the larvae grown in control conditions, whereas in low pH treatments it was reached by only 7–14%. Considering the slow growth rates in the Baltic Sea, a ca. 10% decrease in the larvae reaching the adequate size and developmental stage for successful settling could be critical to the future sustainability of the population.

**Figure 5:**
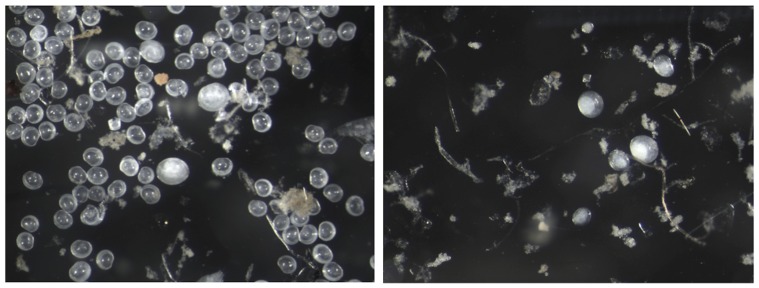
Indication of shell dissolution in different pH treatments. The left picture shows the situation after the 20-day experiment in control conditions, picturing several empty shells of *M. balthica* larvae. The right picture is from the pH_7.2_ at the same time point, with the majority of the dead shells absent, implying a high dissolution of the shell material in low pH conditions.

In addition, at the end of the experiment we found a large amount of empty small *M. balthica* shells in the control treatment, whereas in the reduced pH treatments the majority of the dead small shells were absent (Fig. 5). Calcium carbonate and aragonite saturation states were extremely low (<0.5) in pH_7.2_ and pH_7.4_, and <1 in pH_7.7_. In the control treatment, saturation states were always >1 (Table 1). This implies a fast dissolution of the shell material in low pH conditions. For the organisms living in such conditions, the maintenance of a normal shell structure demands a high energy expenditure, which needs to be balanced with energy allocated to other vital processes. Enhanced dissolution might in fact be a significant threat to organisms, similar to their inability to calcify in low pH conditions [50], [51]. In high pCO_2_ conditions, dissolution of material built of CaCO_3_ has been shown to exceed the rate of calcification, yet with high food availability bivalves may effectively compensate for this [52].

The strong impact of OA on larval growth observed in our study is alarming. Slowly growing larvae face a risk of delayed settling and higher predation risk in response to the longer time they are required to spend in the plankton. The population dynamics of a bivalve species is largely dependent on successful settlement and recruitment of the post-larvae [48], and dispersal of larval and post-larval stages from adjacent areas aid in maintaining a healthy benthic community [53], [54]. The Baltic Sea is severely eutrophied and experiences large-scale hypoxia, the extent of which varies seasonally, with large areas of the sea floor being permanently hypoxic [28]. These hypoxic conditions severely reduce macrofaunal biodiversity [22], [55], and are predicted to increase in amplitude and magnitude in the Baltic Sea over the coming decades [56], leading to even higher risk of degraded benthic communities that are both structurally and functionally impaired. Future survival of key-species, such as *M. balthica* is essential for maintaining important sediment ecosystem functions, such as sediment oxygen- and nutrient cycling [21], [22], [57] and secondary biomass production [58]. The maintenance of such functions is of uttermost importance in disturbance-prone areas like the Baltic Sea.

We observed severe impacts of reduced pH on *M. balthica* larvae, even though the species is assumed to be well-adapted to living in a temporally acidified system such as the Baltic Sea. We predict that even a slight pH decrease can cause significant negative changes during the larval phase of this key bivalve species, both by slowing growth and by decreasing survival. In the Baltic Sea, the pH conditions that are predicted for the future oceans are already regularly occurring. The system might thus work as an analogue for future ocean acidification scenarios.
